# Liposomal Formulations Loaded with a Eugenol Derivative for Application as Insecticides: Encapsulation Studies and In Silico Identification of Protein Targets

**DOI:** 10.3390/nano12203583

**Published:** 2022-10-13

**Authors:** Maria José G. Fernandes, Renato B. Pereira, Ana Rita O. Rodrigues, Tatiana F. Vieira, A. Gil Fortes, David M. Pereira, Sérgio F. Sousa, M. Sameiro T. Gonçalves, Elisabete M. S. Castanheira

**Affiliations:** 1 Centre of Chemistry (CQUM), University of Minho, Campus de Gualtar, 4710-057 Braga, Portugal; 2 REQUIMTE/LAQV, Laboratory of Pharmacognosy, Department of Chemistry, Faculty of Pharmacy, University of Porto, R. Jorge Viterbo Ferreira, 228, 4050-313 Porto, Portugal; 3 Physics Centre of Minho and Porto Universities (CF-UM-UP), University of Minho, Campus de Gualtar, 4710-057 Braga, Portugal; 4 Associate Laboratory LaPMET, University of Minho, Campus de Gualtar, 4710-057 Braga, Portugal; 5 UCIBIO/REQUIMTE, BioSIM—Department of Medicine, Faculty of Medicine, University of Porto, Alameda Prof. Hernâni Monteiro, 4200-319 Porto, Portugal; 6 Associate Laboratory i4HB—Institute for Health and Bioeconomy, Faculty of Medicine, University of Porto, 4200-319 Porto, Portugal

**Keywords:** eugenol derivatives, nanoencapsulation, liposomal formulations, inverted virtual screening, protein targets, insecticides

## Abstract

A recently synthesized new eugenol derivative, ethyl 4-(2-methoxy-4-(oxiran-2-ylmethyl)phenoxy)butanoate, with a high insecticidal activity against *Sf9* (*Spodoptera frugiperda*) insect cells, was encapsulated in the liposomal formulations of egg-phosphatidylcholine/cholesterol (Egg-PC:Ch) 70:30 and 100% dioleoylphosphatidylglycerol (DOPG), aiming at the future application as insecticides. Compound-loaded DOPG liposomes have sizes of 274 ± 12 nm, while Egg-PC:Ch liposomes exhibit smaller hydrodynamic diameters (69.5 ± 7 nm), high encapsulation efficiency (88.8 ± 2.7%), higher stability, and a more efficient compound release, thus, they were chosen for assays in *Sf9* insect cells. The compound elicited a loss of cell viability up to 80% after 72 h of incubation. Relevantly, nanoencapsulation maintained the toxicity of the compound toward insect cells while lowering the toxicity toward human cells, thus showing the selectivity of the system. Structure-based inverted virtual screening was used to predict the most likely targets and molecular dynamics simulations and free energy calculations were used to demonstrate that this molecule can form a stable complex with insect odorant binding proteins and/or acetylcholinesterase. The results are promising for the future application of compound-loaded nanoliposome formulations as crop insecticides.

## 1. Introduction

The synthesis of new bioinspired insecticides is of huge relevance in agriculture, considering that these compounds can eradicate pests with high activity and low toxicity, being eco-friendly substituents of synthetic insecticides [1].

Eugenol, a volatile phenylpropanoid constituent of clove essential oil found in *Eugenia caryophyllata* (*Syzygium aromaticum*) buds and leaves, is a functional ingredient of a broad range of products used in the pharmaceutical, food, and cosmetic industries, as well as dentistry and agriculture, in controlled concentrations [2]. The importance of eugenol is related to several relevant biological activities that it possesses, namely as an analgesic [3], anti-inflammatory [4], hypotensive [5], anticarcinogenic [6], antioxidant [7], antiseptic, dental analgesic [8], antiparasitic [9], antiviral [10], leishmanicidal [11], antifungal [12], and antimicrobial [13].

Eugenol also exhibits a potent insecticidal activity, and the synthesis of new derivatives can be an important way to modulate the biological activity of these compounds [14]. A set of *O*-alkylated eugenol (trivial name for 4-allyl-2-methoxyphenol) derivatives, having a propyl chain with terminals such as hydrogen, hydroxyl, ester, chlorine, and carboxylic acid, and their later epoxidation to give the corresponding oxiranes, was previously published by our research group [15]. All derivatives were evaluated against their effect on the viability of insect cell line *Sf9* (*Spodoptera frugiperda*), evidencing that structural changes elicit marked effects in terms of potency [15].

The application of an integrated molecular modelling—inverted virtual screening protocol allowed the identification of potential protein targets for the eugenol derivatives previously reported. The protocol included the study of protein targets typically associated with insecticide activity and five different scoring functions from popular docking software alternatives. Two eugenol derivatives, *O*-alkylated oxiranes bearing the propyl chain with ester and chloride as terminals, turned out to be very promising for future applications as active ingredients in insecticide formulations, with equally important low toxicity towards human cells after 24 h treatment [15]. In order to understand whether the impacts of these two eugenol oxiranes, namely, ethyl 4-(2-methoxy-4-(oxiran-2-ylmethyl)phenoxy)butanoate **1** and 2-(4-(3-chloropropoxy)-3-methoxybenzyl)oxirane **2** were time-dependent, the cytotoxic effects to *Sf9* insect cells were evaluated after 72 h exposure. Additional in silico assays were performed to predict possible targets for these eugenol derivatives. Through an inverted virtual screening approach, 23 common pesticide targets were screened, and the top two targets predicted were further evaluated through molecular dynamics simulations and free energy calculations to further understand the origin of the insecticidal activity of the studied compounds.

Bearing in mind future applications as an insecticide in crops, and in order to circumvent some common limitations of nature-inspired pesticides which hinder their real application (such as reduced stability, volatility, low water solubility, poor bioavailability, and low resistance to the presence of oxygen and light), the most active compound (compound **1**) was loaded into liposomal formulations based on phospholipids and cholesterol, i.e., Egg-PC:Ch (7:3) and 100% DOPG. In fact, encapsulation techniques have arisen as suitable strategies for the preservation of biopesticides [16,17]. More specifically, lipid-based carriers have been widely used as vehicles for cosmetic/pharmaceutical bioactives, plant extracts, and pesticides [18,19,20,21,22]. The best formulation in terms of compound encapsulation efficiency, size, and stability was chosen for assays in *Sf9* insect cell lines, showing the preservation of compound activity. The results obtained point out the promising application of these nanosystems in future eugenol-based insecticide formulations.

## 2. Materials and Methods

### 2.1. Cell Culture

*Sf9* (*Spodoptera frugiperda*, ATCC, VI, USA) cells were maintained as a suspension culture and cultivated in Grace’s medium (Gibco, ThermoFisher Scientific, Waltham, MA, USA) with 10% fetal bovine serum (FBS, Sigma-Aldrich, St. Louis, MA, USA) and 1% penicillin/streptomycin, at 28 °C. Cells were used in experiments while in the exponential phase of growth. HaCaT (human keratinocyte) cells were cultured in Dulbecco’s Modified Eagle Medium (DMEM), Sigma-Aldrich, St. Louis, MA, USA) supplemented with 10% FBS and 1% penicillin/streptomycin at 37 °C, in a humidified atmosphere of 5% CO_2_.

### 2.2. Viability Assessment

Compounds **1** and **2** were prepared in DMSO, in stocks of 40 mg/mL. For the assessment of the viability, a resazurin-based method was used, as previously described [23]. The *Sf9* and HaCaT cells were plated at a density of 1.5 × 10^4^ and 5.0 × 10^3^ cells/well, respectively, incubated for 24 h, and then exposed to the molecules under study (at 6.25–100 µg/mL; maximum DMSO concentration: 0.25%) for 72 h. After this period, a commercial resazurin-based solution, PrestoBlue^TM^ cell viability reagent (Invitrogen, Grand Island, NE, USA), was added (1:10), and the kinetic reaction of fluorescence increase was monitored at 560/590 nm. For HaCaT and the *Sf9* cells, 30 and 60 min of incubation were used, respectively.

### 2.3. Inverted Virtual Screening (IVS) Studies

A search on Scopus was performed for papers describing virtual screening (VS) studies involving targets and molecules with insecticidal activity. The selection criteria were the relevance of the target and the year of publication. In the seventeen studies found, thirteen targets and twenty-three crystallographic structures were identified and are listed in Table 1.

Each target was prepared for IVS using PyMol (Schrodinger, LLC, New York, USA) [41]. The crystallographic ligands, when present, were extracted from the respective targets and saved for binding site reference and posterior validation. When no ligand was present, the binding site was inferred from the bibliography, and the most important amino acid residues were considered. To validate the protocol, re-docking was used. This consists of removing the crystallographic ligand and re-docking it with the docking scoring functions (SFs) to evaluate their ability to reproduce the orientation and position of the ligand. The results are measured in terms of the root mean square deviation (RMSD) between the predicted pose and the reference position in the crystallographic structure, with a value below 2 Å being a measure of good protocol quality.

For this study, five scoring functions were used: PLP, ASP, ChemScore, GoldScore (all part of the GOLD (Cambridge Crystallographic Data Centre, Cambridge, UK, [42] software), and AutoDock Vina (Center for Computational Structural Biology (CCSB), La Jolla, CA, USA) [43]. The docking conditions were equivalent across all the SFs to ensure consistency and reproducibility. The conditions that performed the best were then applied at the IVS stage. The optimized parameters consisted of docking coordinates and box dimension (or radius in the case of GOLD), number of runs, and exhaustiveness or search efficiency.

The chemical structure of the eugenol derivative **1** was prepared using DataWarrior (Actelion/Idorsia Pharmaceuticals Ltd., Allschwil, Switzerland) [44] and OpenBabel (Open source) [45]. Compound **1** was docked into each target with all the SF and a list of ranked scores was created. This protocol is well established and has been applied to other IVS studies involving other eugenol and carvacrol derivatives [23,46].

### 2.4. Molecular Dynamics Simulations and Free Energy Calculations

The IVS predictions were confirmed by performing molecular dynamics simulations (MD) on the most promising targets predicted: Acetylcholinesterase (PDB: 1QON) and Odorant binding protein 1 (PDB: 3K1E). Since for MD simulations the protein structures must not present any gaps, a homology model was created using SWISSMODEL (Biozentrum, University of Basel, Basel, Switzerland) [47] (Figure S1 in Supplementary Material). The ligand poses used in the MD simulations were the ones predicted with the GOLD/PLP scoring function, posteriorly treated with the Leap module of AMBER (University of California, Los Angeles, CA, USA) [48]. The predicted targets, 1QON and 3K1E, were treated with the ff14SB force field [49] and compound **1** was parameterized using ANTECHAMBER, with the General Amber Force Field (GAFF) [50], with the RESP HF/6-31G(d) charges calculated with Gaussian16 (Gaussian, Inc., Wallingford, CT, USA) [51]. Sodium counter ions (Na^+^) were added to neutralize the charge of the system and the protein-ligand complexes were placed in a TIP3P water box with a 12 Å distance between the surface of the protein and the side of the box, with periodic boundary conditions. The long-range electrostatic interactions were calculated using the particle mesh Ewald summation method. The cut-off value for the short-range electrostatic and Lennard–Jones interactions was set at 10.0 Å. The SHAKE algorithm was used to constrain the hydrogen bonds and a time step of 2 fs was employed.

In this MD protocol, the 100 ns production run was preceded by four minimization steps and two equilibration steps. The four consecutive minimization stages were applied to remove clashes and were applied to the following groups of atoms: 1-water molecule (2500 steps); 2-hydrogens atoms (2500 steps); 3-side chains of all the amino acid residues (2500 steps); and 4-full system (10,000 steps). The first equilibration was performed in an NVT ensemble where the systems were heated to 298 K by applying a Langevin thermostat at a constant volume (50 ps). In the second equilibration step, the density of the systems was further equilibrated at 298 K (subsequent 50 ps). Finally, the production run was performed using an NPT ensemble at constant temperature (298 K) and pressure (1 bar, Berendsen barostat).

The final trajectory obtained for each target was analyzed using the CPPTRAJ tool [52] of AMBER and VMD (NIH Center for Macromolecular Modeling and Bioinformatics, University of Illinois, Urbana, IL, USA) [53]. RMSD, the number of hydrogen bonds formed, and accessible surface area were the parameters calculated to evaluate the stability of the protein-ligand complexes. This overall procedure is robust and has been previously used with success in the treatment of several biomolecular systems [54,55,56,57].

The Molecular Mechanics—Generalized Born Surface Area (MM-GBSA) method [58] was applied to present an estimation of the binding free energies of compound **1** when in complex with acetylcholinesterase and the odorant binding protein 1. A salt concentration of 0.100 mol.dm^−3^ was used and the contribution of the amino acid residues was accessed by applying the energy decomposition method to each complex. From each MD trajectory, a total of 1400 conformations taken from the last 70 ns of the simulation were considered for the MM-GBSA calculations.

### 2.5. Nanoencapsulation Studies and Release Assays

**Figure 1:**
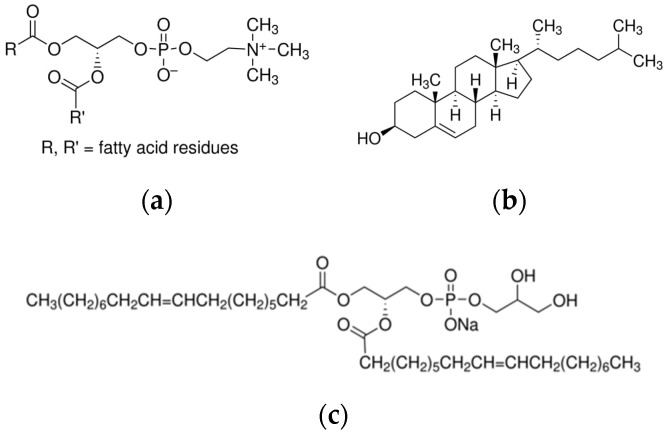
Structure of the lipids used in the liposomal formulations. (**a**) Egg phosphatidylcholine, (**b**) Cholesterol, and (**c**) Dioleoylphosphatidylglycerol.

Compound **1**, ethyl 4-(2-methoxy-4-(oxiran-2-ylmethyl)phenoxy)butanoate, was used for nanoencapsulation studies in the liposomes of 1,2-diacyl-*sn*-glycero-3-phosphocholine from egg yolk (egg phosphatidylcholine, Egg-PC)/cholesterol (Ch) (70% Egg-PC, 30% Ch) and of 100% 1,2 dioleoyl-*sn*-glycero-3-[phospho-*rac*-(1-glycerol)] (dioleoylphosphatidylglycerol, DOPG) (from Sigma-Aldrich, St. Louis, MO, USA) (lipid structures in Figure 1).

The ethanolic injection method was employed to prepare the nanoliposomes. To do so, an ethanolic solution of lipids and compound **1** was slowly injected into an aqueous solution under vortexing [59]. The encapsulation efficiency, *EE(*%), was obtained through Equation (1),

(1) $$EE\left(\%\right)=\frac{Total\;amount-Amount\;of\;nonencapsulated\;compound}{Total\;amount}\times 100$$

The amount of non-encapsulated compound was isolated from the compound-loaded liposomes resorting to Amicon® Ultra centrifugal filter units of 100 kDa (Sigma-Aldrich, St. Louis, MA, USA) by centrifugation at 3000 rpm for 10 min. After centrifugation, the filtrate (containing the non-encapsulated compound) was pipetted out and the fluorescence spectrum was measured. The maximum fluorescence intensity allowed us to determine the concentration of the non-encapsulated compound, using a previously obtained calibration curve (fluorescence intensity vs. concentration). Fluorescence spectra were measured in a Fluorolog 3 spectrofluorometer (HORIBA Jobin Yvon IBH Ltd., Glasgow, UK) and three independent assays were carried out for the determination of the encapsulation efficiency in each liposomal formulation. The UV-Vis absorption spectrum of compound **1** was obtained in a Shimadzu UV-3600 Plus UV-Vis-NIR spectrophotometer (Shimadzu Corporation, Kyoto, Japan).

The structural characterization and stability of liposomes were evaluated by Dynamic Light Scattering (DLS), using a Litesizer 500 equipment from Anton Paar (Anton Paar GmbH, Graz, Austria) with a solid-state laser of 648 nm and 40 mW. For the hydrodynamic diameter and zeta potential measurements of the compound-loaded liposomes, three independent measurements (at 25 °C) were performed.

Release assays from the compound-loaded liposomes were carried out using Amicon^®^ Ultra-0.5 mL centrifugal filters with 0.1 µm pore size. The upper part of the filter was filled with the compound-loaded liposomes, while the bottom was filled with a phosphate buffer solution (pH = 7). At the time of the experiment, 200 µL aliquots were collected from the bottom compartment, and an equal volume of fresh buffer was added to determine the cumulative compound release. The fluorescence intensity of the aliquots was measured, and the release kinetics was fitted to the first-order kinetic model [60] and Weibull model [61].

The first-order kinetic model follows Equation (2),

(2) $$F\left(\%\right)=M_{0}\times \left(1-e^{-kt}\right)$$

where *F*(%) is the percentage of the released compound, *M*_0_ represents the total amount of compound released, *k* represents the first-order rate constant, and *t* is time.

The Weibull model is a distribution function, which expresses the compound fraction accumulated (*m*) in solution at time t, following Equation (3),

(3) $$m=1-\exp\left[\frac{-{\left(t-T_{i}\right)}^{b}}{a}\right]\;$$

where *a* is a parameter defining the timescale of the process, *T_i_* is a location parameter representing the latency time of the release mechanism (considered zero many times), and *b* denotes the curve type shape parameter. For *b* > 1, the transport follows a complex release mechanism; *b* ≤ 0.75 indicates Fickian diffusion (in either fractal or Euclidian spaces) and 0.75 < *b* < 1 indicates a combined mechanism (Fickian diffusion and Case II transport) [61].

### 2.6. Statistical Analysis

For biological assays, the Shapiro–Wilks normality test was performed on the data to ensure that it followed a normal distribution. Cell viability, the comparison between the mean of control and each experimental condition (tested concentrations of compound **1** or **2**), was performed using ordinary one-way ANOVA—Dunnett’s multiple comparisons test; comparison between the means of compound **1** and compound **1**-loaded liposomes was done using two-way ANOVA—Sidak’s multiple comparisons test. Outliers were identified by the Grubbs test. Data were expressed as the mean ± standard deviation (SD) of at least three independent experiments. GraphPad Prism 8.0 software was used, and values were considered statistically significant when *p* < 0.05.

## 3. Results and Discussion

### 3.1. Toxicity of Eugenol Derivatives towards Insect Cells Have a Time-Dependent Effect

**Figure 2:**
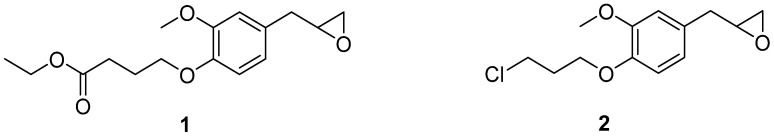
Structure of the eugenol derivatives ethyl 4-(2-methoxy-4-(oxiran-2-ylmethyl)phenoxy)butanoate **1** and 2-(4-(3-chloropropoxy)-3-methoxybenzyl)oxirane **2**.

Ethyl 4-(2-methoxy-4-(oxiran-2-ylmethyl)phenoxy)butanoate **1** and 2-(4-(3-chloropropoxy)-3-methoxybenzyl)oxirane **2** (Figure 2) were obtained by epoxidation with m-chloroperbenzoic acid in dichloromethane at room temperature of ethyl 4-(4-allyl-2-methoxyphenoxy)butanoate and 4-allyl-1-(3-chloropropoxy)-2-methoxybenzene, respectively, which resulted from the *O*-alkylation of eugenol, the trivial name for 4-allyl-2-methoxyphenol, with ethyl 4-bromobutanoate or 1-bromo-3-chloro-propane, using cesium carbonate in acetonitrile, at 65 °C. The compounds presented a purity higher than 95%, according to the ^1^H NMR spectra [15]. The stability of these compounds was tested by ^1^H NMR, and no structural changes were detected during storage at low temperatures (0–5 °C) over a period of 24 months.

**Figure 3:**
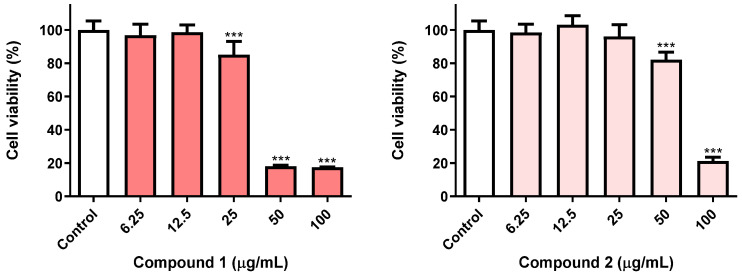
Viability of *Sf9* cells exposed to the molecules **1** and **2** (6.25–100 µg/mL), medium (control). Cells were incubated for 72 h, after which, viability was evaluated. *** *p* < 0.001.

Continuing our ongoing research on the insecticidal properties of eugenol-based molecules [15,23], we were particularly interested in exploring whether the impacts of oxiranes **1** and **2** on *Sf9* insect cell viability were time-dependent. With this aim in view, *Sf9* cells were treated for 72 h with both molecules, at five different concentrations (6.25–100 µg/mL) and compared with the results previously reported after 24 h exposure [15]. As shown in Figure 3, the eugenol derivatives **1** and **2**, after 72 h treatment, caused ca. 80% loss of insect cell viability at 100 µg/mL, a result markedly different than that we have found at 24 h (ca. 50 and 60% viability loss for compound **1** and **2**, respectively) [15]. Furthermore, compound **1** at 72 h displayed the capacity to trigger a similar effect even at 50 µg/mL, being more toxic to *Sf9* insect cells than compound **2**. For this reason, the eugenol derivative **1** was chosen for further computational studies and nanoencapsulation assays in liposomal formulations, keeping in mind a future application as an insecticide.

### 3.2. Inverted Virtual Screening Results

The score obtained for each SF, for compound **1** in the complex of all the possible targets, is depicted in Table 2. The range of values is different because the SFs are based on different metrics and scales. The GOLD SF is dimensionless, and a more positive value indicates a better binding affinity. AutoDock Vina, on the other hand, uses a system of measurement that is a more realistic approximation of the binding free energy, with a more negative score suggesting better affinity. The results were ranked from best to worst. The PDB structure of each set of targets that presented the best score was selected as a potential target. Compound **1** showed increased affinity toward acetylcholinesterase (AChE) and odorant binding protein 1 (OBP). The same tendency was observed for all the independent SF tested, reinforcing these conclusions.

### 3.3. Molecular Dynamics Simulations and Free Energy Calculations Results

**Figure 4:**
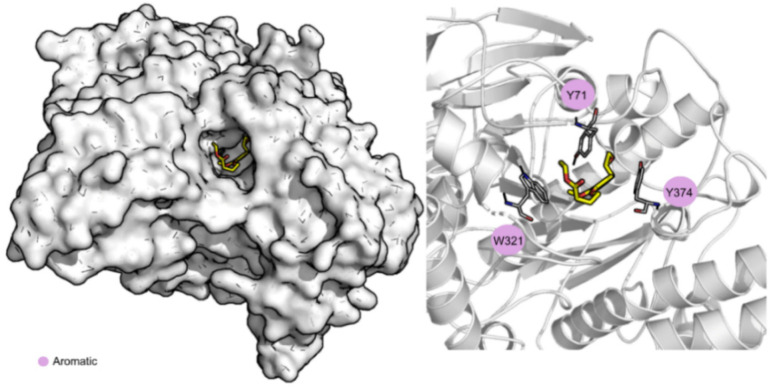
Compound **1** bound to AChE. Surface and cartoon representation. Compound **1** is represented in yellow licorice.

To evaluate the stability of the interactions formed between compound **1** and the two most probable targets predicted, AChE and OBP1, MD simulations were performed using the structure with the best score of these two groups, 1QON for AChE and 3K1E for OBP1. RMSD, solvent accessible surface area (SASA), and the number of hydrogen bonds were the parameters calculated to evaluate the results and are depicted in Table 3. Overall, all the complexes and ligands present a low RMSD value, indicating that the systems are well-equilibrated and stable. Compound **1** is buried into the pocket of both OBP1, with a percentage of potential ligand SASA buried values of 88% and a low ligand SASA (Figure S3 in Supplementary Material). When bound to AChE, it is more exposed to the solvent (percentage of potential ligand SASA buried values of 79%). This indicates that compound **1**, when in complex with OBP1, is highly protected from the solvent and well bound to the protein throughout the simulation. Compound **1** is more exposed to the solvent, throughout the simulation, and when in complex with AChE, mainly due to the exposure of the carboxylic portion of the molecule (as evidenced in Figure 4).

Hydrogen bond analysis allows the understanding of the interactions that occur between compound **1** and the possible targets throughout time. Globally, this compound maintains 0–1 hydrogen bonds, on average, with both OBP1 and AChE (Figure S4 in Supplementary Material).

**Figure 5:**
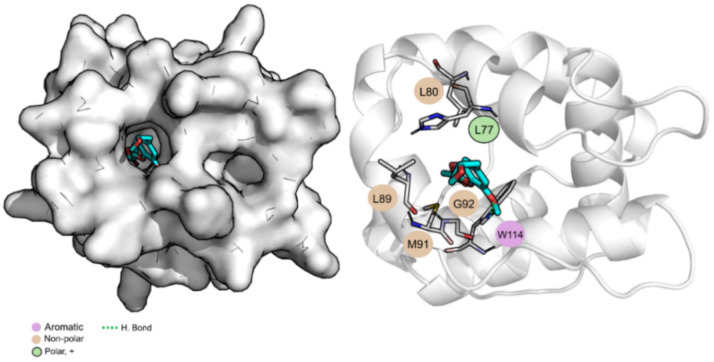
Compound **1** bound to OBP1. Surface and cartoon representation. Compound **1** is represented in cyan licorice.

Table 3 also shows the values for the Gibbs binding free energy of association calculated using MM/GBSA and highlights the three most important amino acid residues involved in the stabilization of compound **1**. The average structure of the dominant cluster of AChE and OBP1 in complex with compound **1** is displayed in Figure 4 and Figure 5, respectively. These figures illustrate the details of the binding pocket and the interaction formed between the targets and compound **1**.

Analyzing the Gibbs binding free energy of association, the results suggest that compound **1** has a higher affinity towards OBP1 than towards AChE (−32.4 kcal/mol vs. −22.6 kcal/mol). When bound to AChE, the compound is mainly stabilized through non-polar interactions with three aromatic residues, Tyr71 (−2.3 ± 0.9 kcal/mol), Trp321 (−1.6 ± 1.0 kcal/mol), and Tyr374 (−1.5 ± 0.8 kcal/mol). Regarding OBP1, compound **1** is stabilized through non-polar interactions with Met91 (−1.3 ± 0.5 kcal/mol) and Gly92 (−1.8 ± 0.6 kcal/mol) but can form a hydrogen bond with Trp114 (−2.5 ± 0.6 kcal/mol). Considering all the data presented, compound **1** seems to be a good candidate to be used as a repellent with OBP1 as its principal target.

### 3.4. Nanoencapsulation Assays in Liposomal Formulations

Compound **1** was encapsulated into liposomal formulations of Egg-PC:Ch (7:3) and DOPG (100%), aiming at obtaining a high encapsulation efficiency and effective release. The ethanolic injection method was chosen considering previous results of release profiles of eugenol, carvacrol, and thymol derivatives [23,46]. The lipid components of the liposomes determine the rigidity, fluidity, and surface charge and, consequently, their ability to be loaded with bioactive compounds and release them [62]. For instance, the use of unsaturated lipids, such as phosphatidylcholines from natural sources (egg or soy lecithin), results in relatively permeable liposomes [63]. On the other hand, cholesterol molecules are also known to modulate membrane rigidity properties. Liposomes of Egg-PC:Ch (7:3) have been used as bilayer models in membrane permeation assays [64,65]. DOPG, the main component of pulmonary surfactant, forms negatively-charged liposomes in a liquid-crystalline phase due to its low transition temperature of −18 °C, originating fluid membranes [66,67].

Prior to nanoencapsulation studies, the absorption and fluorescence emission of eugenol derivative **1** in solution were measured and the corresponding spectra are displayed in Figure S5 in Supplementary Material. An absorption maximum at 280 nm was observed and a fluorescence band between 290 nm and 390 nm was detected, with maximum emission around 320 nm. These properties allow using compound fluorescence to determine the encapsulation efficiencies and release profiles. High encapsulation efficiencies of compound **1** were obtained (Table 4), showing that both formulations are able to encapsulate this molecule at concentrations that may guarantee biological activity. Nonetheless, the liposomes of Egg-PC:Ch (70:30) have been shown to be more efficient to encapsulate this eugenol derivative, presenting higher encapsulation efficiency than DOPG liposomes.

**Figure 6:**
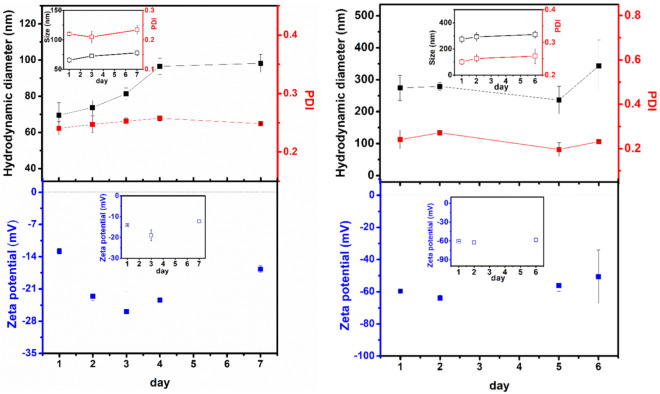
The hydrodynamic diameter (black symbols), polydispersity index (PDI) (red symbols), and zeta potential (blue symbols), of Egg-PC:Ch (7:3) (**left**) and 100% DOPG (**right**) compound-loaded liposomes and empty liposomes (insets). Error bars represent SD from three independent measurements. The DLS size distributions were determined by number.

Liposome stability is an important key for their application. Therefore, a stability study of compound-loaded liposomes was performed measuring structural parameters such as the hydrodynamic diameter, polydispersity index (PDI), and zeta potential over time (Figure 6). The values for the corresponding non-loaded nanosystems are also shown for comparison.

In general, loaded liposomes of Egg-PC:Ch and DOPG were shown to be generally stable for 7 and 6 days, respectively. The loaded nanoformulation of Egg-PC:Ch exhibits structures much smaller in size, compared to the DOPG one, in accordance with previous results of liposomal formulations based on Egg-PC [68] (examples of size distributions in Figure S6 of Supplementary Material). It can be observed that the size of loaded and non-loaded nanosystems is similar and with comparable stability. By 7 days, Egg-PC:Ch liposomes’ size increased from 70 nm to ca. 95 nm (Figure 6—left), a tendency also previously observed in these nanosystems [68], due to their almost neutral charge (very slightly negative zeta potential), pointing to some aggregation. Nevertheless, the loaded nanoliposomes maintain a small size and a low polydispersity index over a week; the same tendency is observed after 15 days (hydrodynamic diameter of 116 ± 24 nm with a PDI of 0.25 ± 0.05). Compound **1**-loaded DOPG liposomes display much larger hydrodynamic diameters of around 280 nm, increasing in size to 340 nm after 6 days, with a small PDI value. The charge repulsion between the negatively-charged phosphate groups (the glycerol moiety in the lipid polar head is neutral) justifies the larger diameters and the overall tendency for a lower aggregation than that of the Egg-PC:Ch system, due to the strongly negative zeta potential of DOPG liposomes (Figure 6—right).

**Figure 7:**
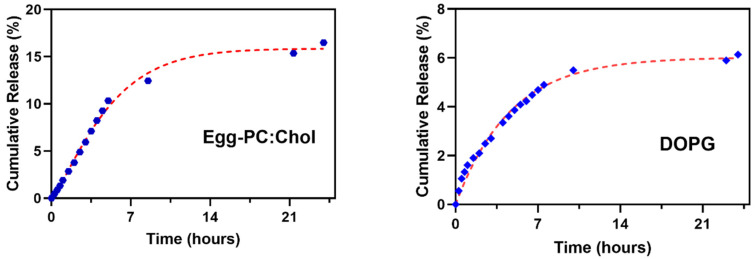
Cumulative release of compound **1** from liposomes of Egg-PC:Cholesterol and DOPG liposomes fitted to the Weibull model.

The cumulative release of eugenol derivative **1** from the two liposomal formulations was assessed for 24 h and the experimental data of release profiles were fitted to the first-order kinetic model (Figure S7 in Supplementary Material) and the Weibull model (Figure 7). The values of the model’s parameters are summarized in Table 5.

Quite similar release profiles were obtained for both liposomal formulations, with an initial burst release followed by a slower decay. Egg-PC:Ch liposomes present a slower compound leakage in the early stage of release than DOPG liposomes. However, after 24 h, the release from DOPG liposomal formulation is very small, showing retention of the compound by these liposomes.

The best-fitting model describing compound **1** release from liposomes was the Weibull model, with higher coefficients of determination for both liposomal formulations (Table 5) and pointing to a compound release by Fickian diffusion (*b* values below 0.75 in both formulations [61]). Nevertheless, the fit to the first-order kinetic model also shows a good correlation (Table 5).

Extended-release assays (72 h) of compound **1** from Egg-PC:Ch liposomes revealed (Figure S8 in Supplementary Material) an effective cumulative release of 80% in three days. Considering the very high encapsulation efficiency (almost 90%) of compound **1** in Egg-PC:Ch liposomes and the release profiles, together with the good stability of the compound-loaded nanosystem, this formulation was chosen for the subsequent assays of insecticidal activity using the compound-loaded nanoliposomes.

### 3.5. Compound **1**-Loaded Egg-PC:Ch Liposomes Maintained Insecticidal Activity with Decreased Toxicity towards Human Cells

**Figure 8:**
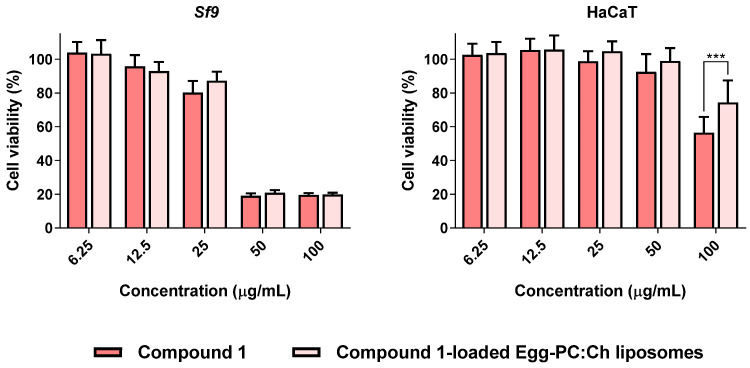
Viability of *Sf9* and HaCaT cells exposed to compound **1** and compound **1**-loaded Egg-PC:Ch liposomes (6.25–100 µg/mL). Cells were incubated for 72 h, after which, viability was evaluated. *** *p* < 0.001.

Considering the encapsulation efficiency and release profile, the liposomes were loaded with compound **1** to obtain the concentration equivalent to 100 µg/mL. To find out if the nanoformulation maintained the cytotoxicity towards *Sf9* insect cells, compound **1**-loaded Egg-PC:Ch liposomes were compared (side-by-side) with compound **1** in another set of experiments (Figure 8, left). With this aim in view, we first explored the putative impact that drug-free liposomes may have on cell viability, and it was proven that they have no effect on the viability of the cells under study (*Sf9* cells and HaCaT cells) (Figure S9 in Supplementary Material). The cytotoxicity of compound **1** and compound **1**-loaded liposomes is depicted in Figure 8. It was proven that the encapsulation of compound **1** in liposomes did not result in a loss of toxicity toward *Sf9* insect cells (Figure 8, left), which is a promising finding. Furthermore, bearing in mind the importance of developing formulations that have a safer toxicological profile and considering the usual routes of pesticide poisoning, specifically skin, cytotoxicity towards keratinocytes (HaCaT cells) was explored. Noteworthy, the encapsulation of compound **1** into Egg-PC:Ch liposomes led to a significant decrease in toxicity towards human cells of nearly 20% at the highest concentration tested (100 µg/mL) (Figure 8, right).

## 4. Conclusions

The compound ethyl 4-(2-methoxy-4-(oxiran-2-ylmethyl)phenoxy)butanoate, a recently synthesized new eugenol derivative with promising insecticidal activity, was encapsulated in the liposomes of Egg-PC:cholesterol (70:30) and 100% DOPG, with its future use in insecticide formulations in mind.

To understand the compound’s insecticidal activity more deeply, the most likely targets were predicted, and the stability of the complex was confirmed using in silico methods, including structure-based inverted virtual screening and molecular dynamics simulations. Free energy calculations were used to estimate the binding free energy and provide a detailed characterization of the binding mode. Insect acetylcholinesterase and/or odorant binding proteins are, most likely, the targets of this compound since the resulting complexes exhibit appropriate predicted binding affinity and are stable throughout the simulation time.

The compound-loaded nanoliposomes were generally stable for 6–7 days and allowed high encapsulation efficiencies, with the Egg-PC:Ch nanocarrier providing a more effective compound release. This formulation was chosen for biological assays, and the results showed that the encapsulation of the compound in liposomes did not result in a loss of toxicity toward insecticidal activity in *Sf9* cells and led to a significant decrease in toxicity toward human cells, specifically in keratinocyte (HaCaT cells) cells with nearly 20% at the highest concentration tested (100 µg/mL). These results are promising findings for the future application of compound-loaded liposomes as insecticidal formulations.

## Figures and Tables

**Table 1:** List of targets selected for the inverted virtual screening study.

Target	Organism	PDB Target	Resolution (Å)	Description	Ref.
**Ecdysone receptor**	*Heliothis virescens*	1R20	3.00	VS based on 1R20 bound to an agonist as a model for the development of a receptor-based pharmacophore model.	[24]
		1R1K	2.90	VS of 2 million compounds against 1R1K, an ecdysone receptor structure bound to its known ligand ponasterone A.	[25]
**Chitinase**	*Ostrinia furnacalis*	3WL1	1.77	Pharmacophore-based screening using two crystal structures of chitinases: 3WL1 bound to its reaction product and 3WQV bound to an inhibitor.	[26]
		3WQV	2.04		
**β-*N*-Acetyl-D-hexosaminidase OfHex1**		3NSN	2.10	VS of the ZINC database to identify OfHex1 inhibitors using 3NSN crystal structure bound to a known inhibitor.	[27]
		3OZP	2.00	VS of the ZINC database targeting 3OZP, a crystal structure of OfHex1 bound to an inhibitor.	[28]
***N*-Acetylgluco-samine-1-phosphate uridyltransferase (GlmU)**	*Xanthomonas oryzae*	2V0K	2.30	Homology model built for docking using 2V0K and 2VD4 as templates. The 2V0K crystal structure is bound to its known ligand and 2VD4 is bound to a possible inhibitor.	[29]
		2VD4	1.90		
**Acetyl-** **cholinesterase**	*Aedes aegypti*	1QON	2.72	Search for new molecules with insecticidal activity against *Ae. Aegypti* using acetylcholinesterase crystal structures 1QON and 4EY6 as targets, both bound to possible inhibitors.	[30]
		4EY6	2.40		
	*Drosophila melanogaster*	1DX4	2.70	Homology 3D model built for VS using 1DX4 as a template. 1DX4 crystal structure is bound to a potent inhibitor.	[31]
**Polyphenol- oxidase**	*Manduca sexta*	3HSS	2.70	Crystal structure of a prophenoloxidase from *Manduca sexta*.	[32]
***p*-Hydroxyphenyl-pyruvate dioxygenase**	*Arabidopsis thaliana*	6ISD	2.40	Development of a receptor-ligand pharmacophore model based on the crystal structure 6ISD bound to a commonly used pesticide. The best model created was then used for VS studies.	[33]
**Voltage-gated sodium channel**	*Periplaneta americana*	6A95	2.60	Crystallographic structure of a voltage-gated sodium channel, NavPaS, bound to a pore blocker, tetrodotoxin (TTX)	[34]
**Octopamine receptor**	*Blattella germanica*	4N7C	1.75	Crystal structure of Bla g 4, an octopamine receptor, bound to tyramine.	[35]
**Sterol carrier protein-2 (HaSCP-2)**	*Helicoverpa armigera*	4UEI	Solution NMR	Structure-based VS of a database of commercially available compounds to find potential inhibitors of HaSCP-2. The residues Phe53, Thr128, and Gln131 were selected for thebinding cavity.	[36]
**Peptide deformylase**	*Xanthomonas oryzae*	5CY8	2.38	Docking and VS of a library of 318 phytochemicals. The 5CY8 crystal structure is bound to a possible inhibitor.	[37]
**Alpha-esterase-7 (αE7)**	*Lucilia cuprina*	5TYJ	1.75	Computational design of potent and selective covalent inhibitors of αE7. The 5TYJ and 5TYP crystal structures are bound to inhibitors: (3-bromo-5-phenoxylphenyl)boronic acid and (3-bromo-4-methylphenyl)boronic acid, respectively.	[38]
		5TYP	1.88		
**Odorant Binding Protein**	*Aedes aegypti*	5V13	1.84	Search for new molecules with insecticidal activity against *Ae. Aegypti* using a crystal structure of a mosquito juvenile hormone-binding protein, 5V13, bound to its natural hormone.	[30]
	*Drosophila melanogaster*	2GTE	1.40	The 2GTE crystal structure is bound to its natural ligand	[39]
	*Anopheles gambiae*	3N7H	1.60	QSAR and docking studies for the rational design of mosquito repellents using the crystal structure 3K1E bound to a polyethylene glycol molecule. The 3N7H crystal structure is bound to a commonly used repellent.	[40]
	*Aedes aegypti*	3K1E	1.85		[40]

**Table 2:** Scoring values of the eugenol derivative **1** obtained for all putative targets PDB structures with the five different scoring functions and overall ranking of the most likely protein targets for interaction.

Target	PDB	PLP	ASP	ChemScore	GoldScore	Vina	Overall Ranking
**Acetylcholinesterase**	**1QON**	81.19	59.26	37.75	69.05	−8.30	1
	**1DX4**	77.08	51.68	36.22	62.54	−7.70	
	**4EY6**	76.18	48.57	32.96	59.66	−7.60	
**Alpha-esterase-7**	**5TYJ**	59.68	33.92	32.74	54.09	−6.20	8
	**5TYP**	63.72	39.54	31.84	56.73	−6.20	
**Beta-N-acetyl-D-hexosaminidase OfHex1**	**3OZP**	71.22	49.42	29.92	63.78	−7.30	4
	**3NSN**	76.67	54.51	32.45	67.93	−6.50	
**Chitinases**	**3WQV**	68.53	49.59	31.24	65.25	−6.60	3
	**3WL1**	70.09	48.98	31.76	60.37	−7.00	
**Ecdysone receptor (EcR)**	**1R1K**	66.83	31.02	32.21	61.61	−7.70	5
	**1R20**	63.97	29.6	29.98	58.93	−6.80	
**N-Acetylglucosamine-1-phosphate uridyltransferase (GlmU)**	**2V0K**	58.12	28.56	23.72	59.67	−5.90	13
	**2VD4**	52.43	25.36	20.76	48.33	−5.20	
**Octopamine receptor**	**4N7C**	60.27	39.29	34.34	69.10	−5.90	7
**Odorant Binding Protein**	**2GTE**	70.46	39.20	30.71	66.39	−6.30	2
	**3K1E**	83.01	44.98	37.85	66.89	−5.90	
	**5V13**	80.20	49.37	40.18	63.84	−7.70	
	**3N7H**	74.95	39.20	30.71	66.39	−6.30	
**Peptide deformylase**	**5CY8**	72.08	30.11	25.48	71.01	−7.00	6
**p-hydroxyphenylpyruvate dioxygenase**	**6ISD**	68.60	33.78	26.22	51.19	−7.00	9
**Polyphenol oxidase (PPO)**	**3HHS**	62.37	34.09	29.03	66.32	−5.70	11
**Sterol carrier protein-2 (HaSCP-2)**	**4UEI**	64.27	32.22	31.10	50.51	−6.20	10
**Voltage-gated sodium channel**	**6A95**	63.20	28.28	21.92	61.46	−6.10	12

**Table 3:** Average protein and ligand RMSD values (Å), average ligand SASA (Å^2^), percentage of potential ligand SASA buried, and the average number of ligand-target hydrogen bonds obtained from the MD simulations. The ΔG binding energy was determined using MM/GBSA and per-residue decomposition, calculated for the last 70 ns of the simulation.

	Average RMSD of the Complex (Å)	Average RMSD of the Ligand (Å)	Ligand SASA (Å^2^)	Percentage of Potential Ligand SASA Buried (%)	Average Number H-Bonds	ΔG_bind_ (kcal/mol)	Main Contributors (kcal/mol)
**AChE**	3.4 ± 0.3	1.7 ± 0.4	111.2 ± 53.2	79	0.1 ± 0.2	−22.6 ± 0.2	Tyr71 (−2.3 ± 0.9)
							Trp321 (−1.6 ± 1.0)
							Tyr374 (−1.5 ± 0.8)
**OBP1**	2.1 ± 0.3	1.2 ± 0.2	63.7 ± 16.3	88	0.01 ± 0.1	−32.4 ± 0.2	Met91 (−1.3 ± 0.5)
							Gly92 (−1.8 ± 0.6)
							Trp114 (−2.5 ± 0.6)

**Table 4:** Encapsulation efficiency, EE(%) ± SD(%), of compound **1** in liposomes (SD: standard deviation).

Liposomes	EE(%) ± SD(%)
Egg-PC:Ch (70:30)	88.8 ± 2.7
DOPG (100%)	79.8 ± 2.6

**Table 5:** Parameters obtained by fitting the release profiles to the first-order kinetic model and Weibull model, and the respective coefficients of determination (*R*^2^).

Liposomes	First-Order Kinetics		Weibull		
	*k*	*R* ^2^	*b*	*a*	*R* ^2^
Egg-PC:Ch (70:30)	0.16	0.988	0.1258	1.244	0.995
DOPG (100%)	0.21	0.985	0.2469	0.8457	0.9904

## Data Availability

Not applicable.

## Supplementary Materials

The following supporting information can be downloaded at: https://www.mdpi.com/article/10.3390/nano12203583/s1, 1. Creation of a Homology Model: Figure S1. Homology model built for 1QON; Figure S2. Protein and ligand RMSD (Å) of the AChE and OBP—ligand complexes; Figure S3. Percentage of the potential solvent accessible surface area of the ligands that are buried by the protein targets evaluated; Figure S4. The number of ligand-target hydrogen bonds formed during the simulations for compound **1** when complexed with AChE and OBP; 2. Encapsulation studies: Figure S5. Normalized absorption and fluorescence emission (excitation at 280 nm) spectra of compound **1** in ethanol (1 × 10^−5^ M for absorption and 1 × 10^−6^ M for emission); Figure S6. Examples of size distributions of compound-loaded liposomes obtained from DLS measurements; Figure S7. Cumulative release (for 24 h) of compound **1** from liposomes of Egg-PC:Cholesterol (left) and DOPG (right) liposomes fitted to the first-order kinetic model; Figure S8. Cumulative release (for 72 h) of compound **1** from liposomes of Egg-PC:Cholesterol; Figure S9. Viability of *Sf9* and HaCaT cells exposed to drug-free liposomes (6.25–100 µg/mL), medium (control).
