# Supplementary material for: Liposomal Formulations Loaded with a Eugenol Derivative for Application as Insecticides: Encapsulation Studies and In Silico Identification of Protein Targets
Source: Nanomaterials (Basel). 2022 Oct 13;12(20):3583. doi: 10.3390/nano12203583 (PMC9611868; doi:10.3390/nano12203583)
Supplement: Supplementary file 1 [file nanomaterials-12-03583-s001.zip › nanomaterials-1961531-supplementary.pdf]

Supplementary Material

Liposomal formulations loaded with a eugenol derivative for application as insecticides: encapsulation studies and in silico identification of protein targets

Maria José G. Fernandes[1], Renato B. Pereira[2], Ana Rita O. Rodrigues[3,4], Tatiana F. Vieira[5,6], A. Gil Fortes[1], David M. Pereira[2], Sérgio F. Sousa[5,6], M. Sameiro T. Gonçalves[1], Elisabete M. S. Castanheira[3,4,*]
* ecoutinho@fisica.uminho.pt

1 Centre of Chemistry, Department of Chemistry, University of Minho, *Campus* of Gualtar, 4710-057 Braga, Portugal.
2 REQUIMTE/LAQV, Laboratory of Pharmacognosy, Department of Chemistry, Faculty of Pharmacy, University of Porto, R. Jorge Viterbo Ferreira, 228, 4050-313 Porto, Portugal.
3 Physics Centre of Minho and Porto Universities (CF-UM-UP), University of Minho, *Campus* of Gualtar, 4710-057 Braga, Portugal.
4 Associate Laboratory LaPMET - Laboratory of Physics for Materials and Emergent Technologies, University of Minho, *Campus* of Gualtar, 4710-057 Braga, Portugal.
5 UCIBIO/REQUIMTE, BioSIM – Department of Medicine, Faculty of Medicine, University of Porto, Alameda Prof. Hernâni Monteiro, 4200-319 Porto, Portugal.
6 Associate Laboratory i4HB - Institute for Health and Bioeconomy, Faculty of Medicine, University of Porto, 4200-319 Porto, Portugal.

1. Creation of a Homology Model

The model generated by SWISS-MODEL for 1QON was used in the MD simulations since the gap that was missing from the original structure was distant from the active site.

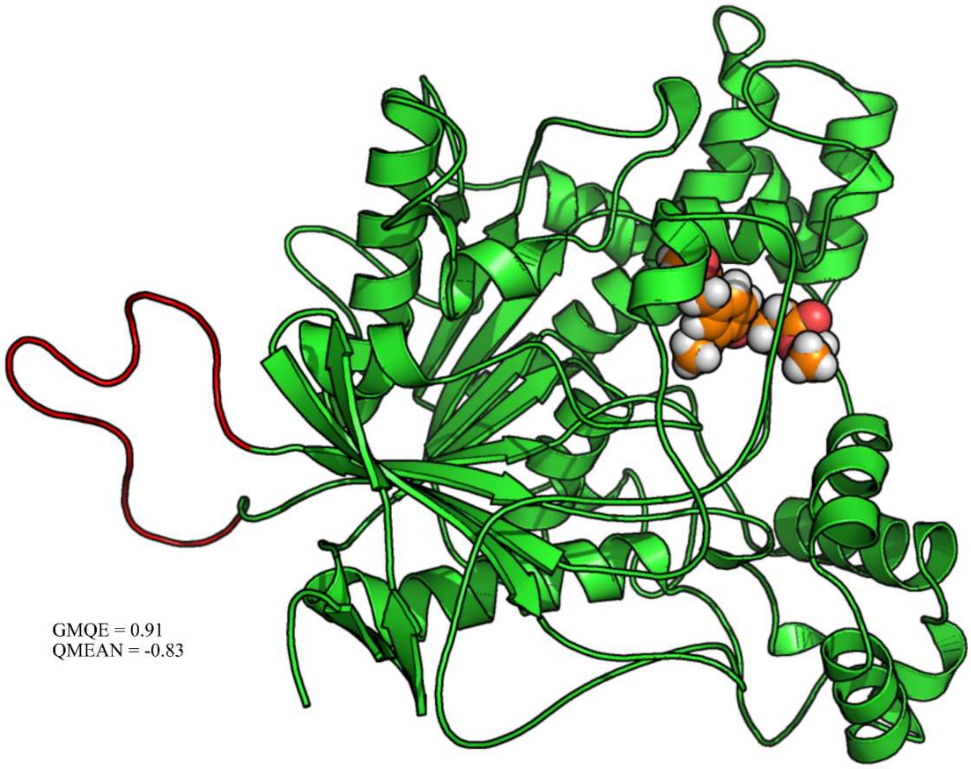

Figure S1. Homology model built for 1QON. Green is the original structure and red represents the loop that was generated by SWISS-MODEL. In orange is the ligand molecule (compound **1**). GMQE - Global Model Quality Estimation, is expressed between 0 and 1 with a higher number meaning higher reliability. QMEAN - provides an estimate of the "degree of nativeness" of the structural features observed in the model. A value of QMEAN around zero indicate a good agreement between the model and experimental structure.

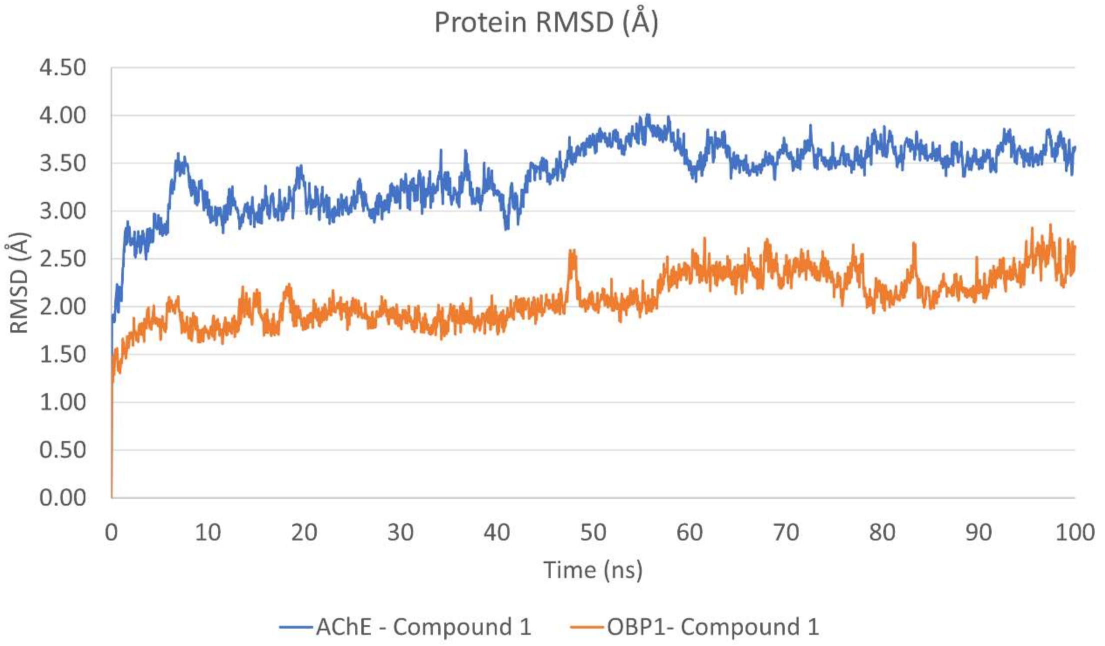

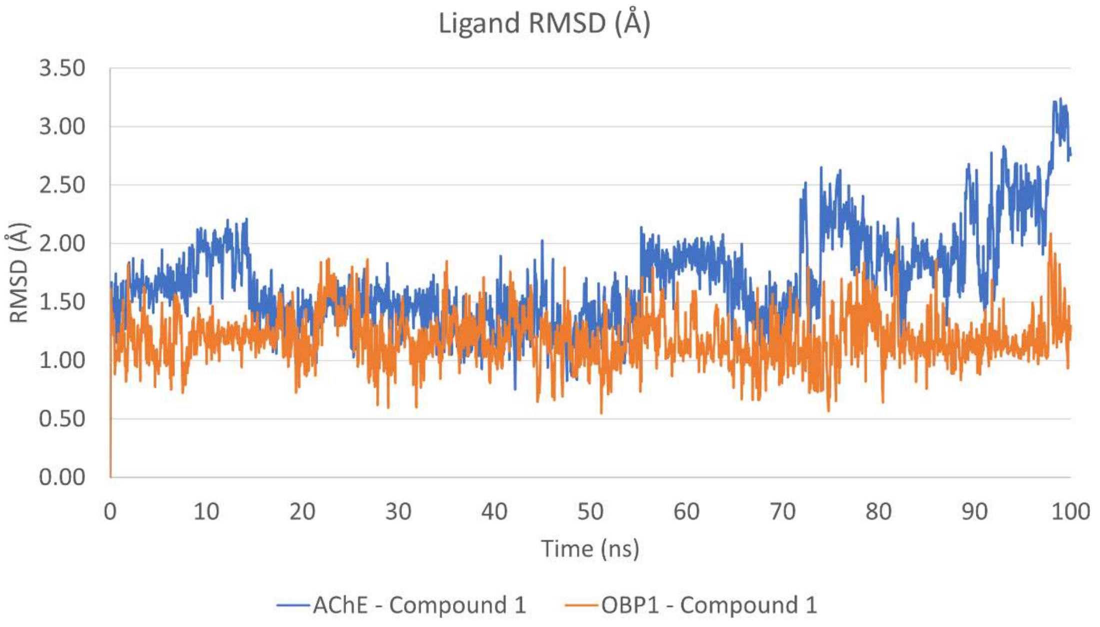

Figure S2. Protein and ligand RMSD (Å) of the AChE and OBP – ligand complexes.

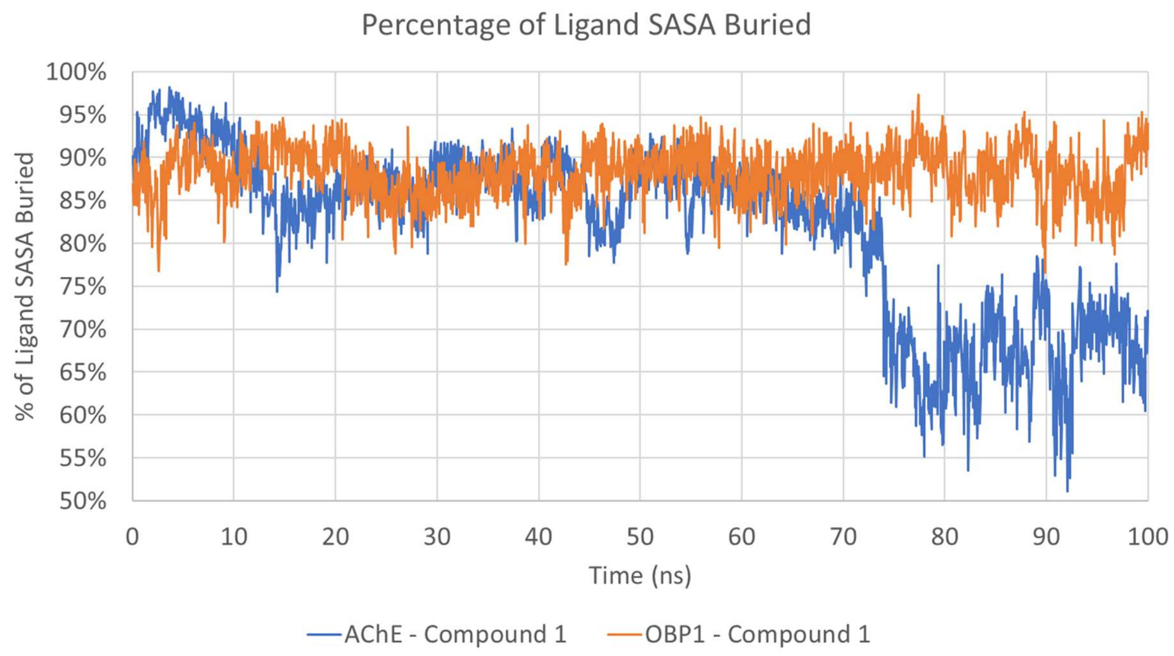

Figure S3. Percentage of the potential solvent accessible surface area of the ligands that is buried by the protein targets evaluated.

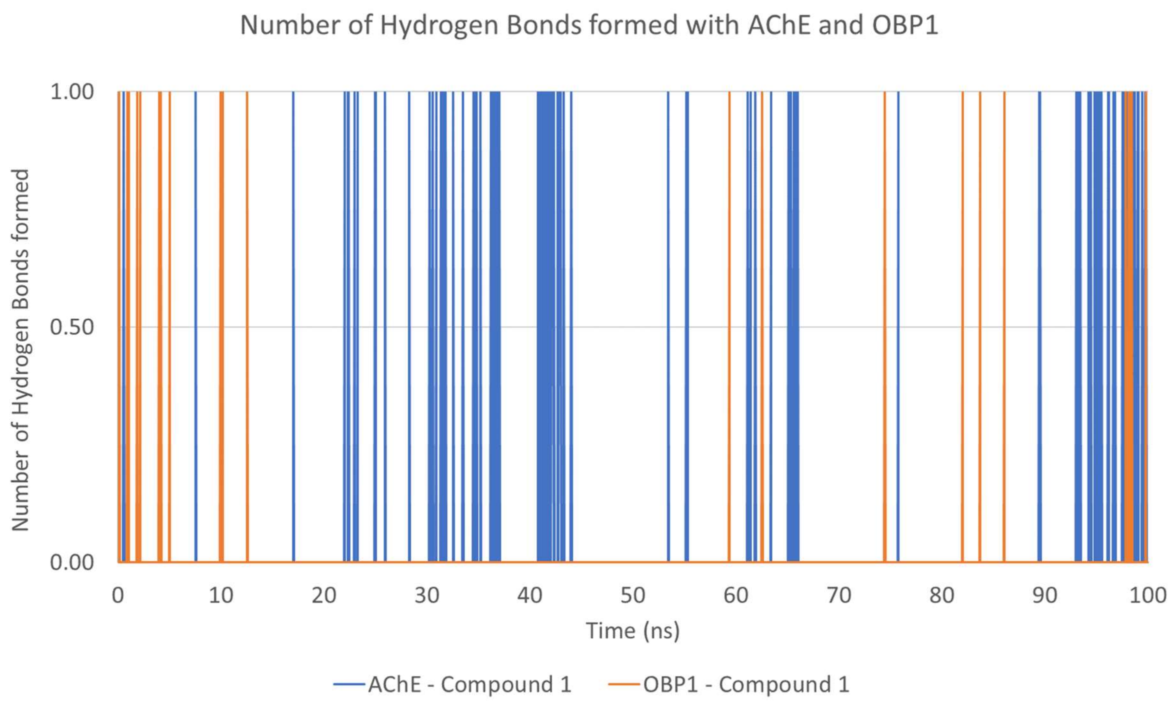

Figure S4. Number of ligand-target hydrogen bonds formed during the simulations for compound **1** when complexed with AChE and OBP.

2. Encapsulation studies

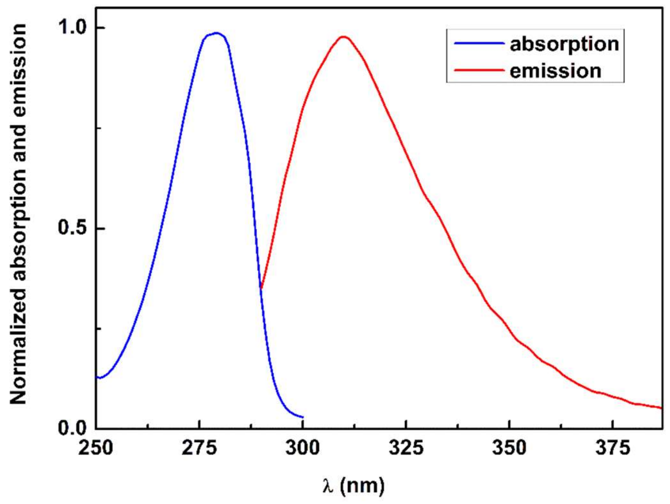

Figure S5. Normalized absorption and fluorescence emission (excitation at 280 nm) spectra of compound **1** in ethanol (1×10^{-5} M for absorption and 1×10^{-6} M for emission).

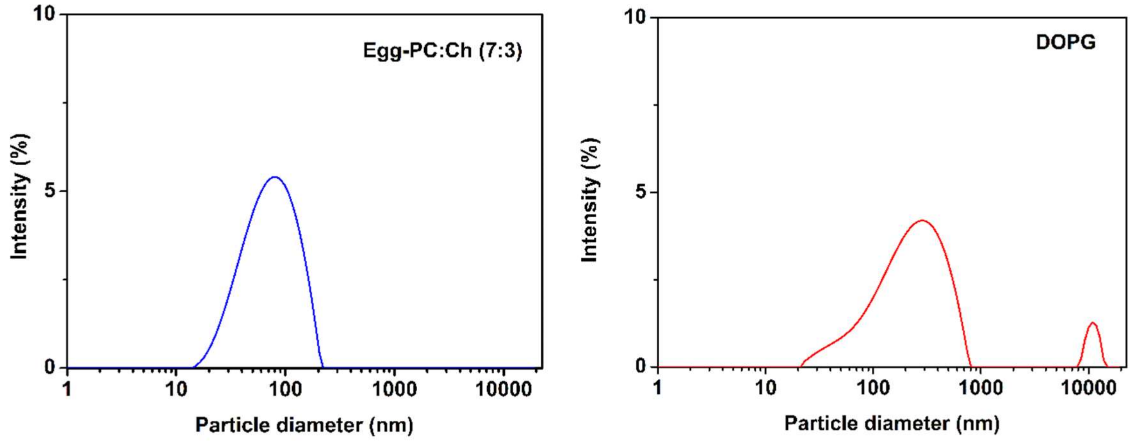

Figure S6. Examples of size distributions of compound-loaded liposomes obtained from DLS measurements.

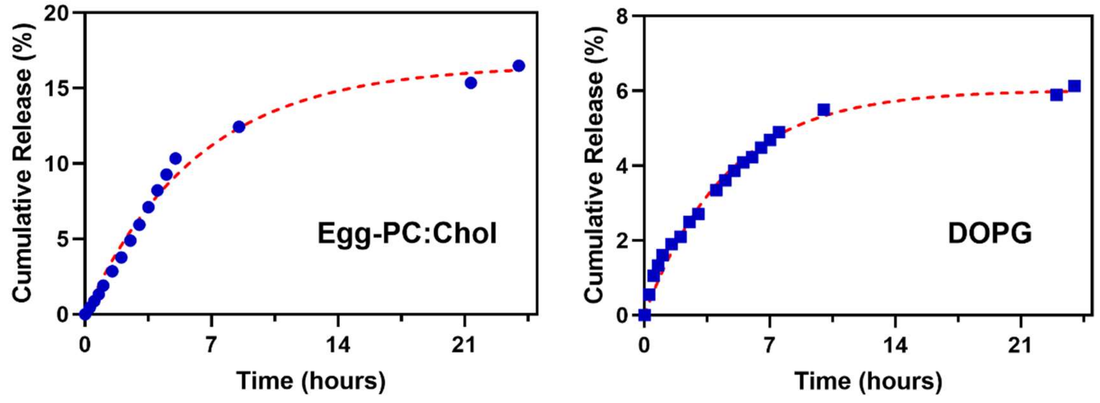

Figure S7. Cumulative release (for 24 h) of compound **1** from liposomes of Egg-PC:Cholesterol (left) and DOPG (right) liposomes fitted to the first-order kinetic model.

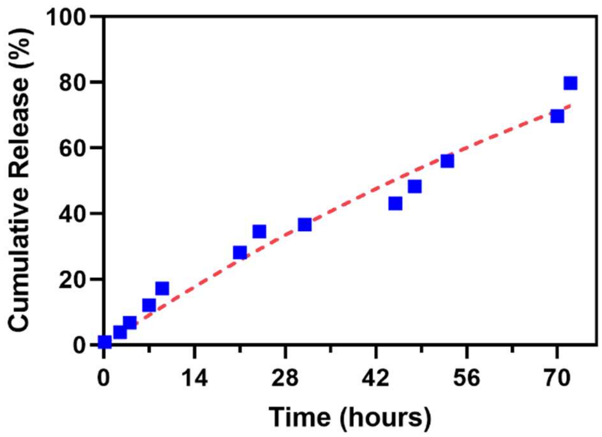

Figure S8. Cumulative release (for 72 h) of compound **1** from liposomes of Egg-PC:Cholesterol.

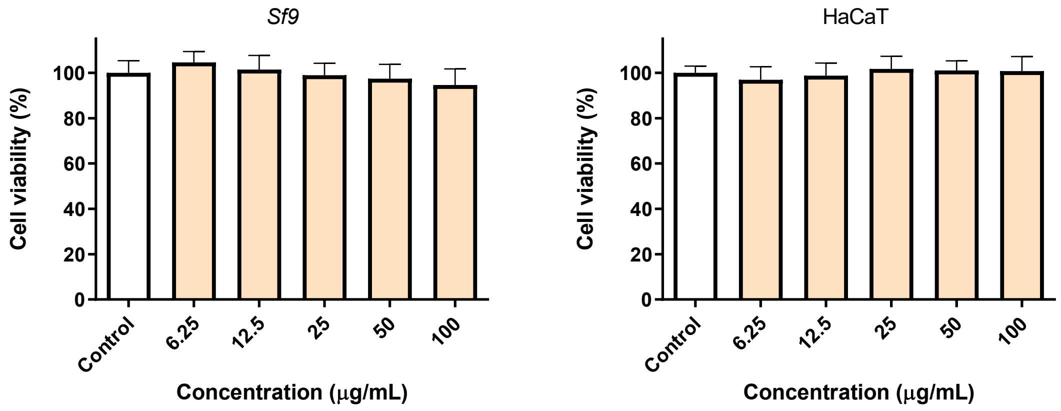

Figure S9. Viability of *Sf9* and HaCaT cells exposed to drug-free liposomes (6.25 – 100 μg/mL), medium (control). Cells were incubated for 72 h, after which viability was evaluated.
